# Gastric cancer secondary prevention in dyspeptic patients

**DOI:** 10.1007/s10120-026-01732-z

**Published:** 2026-04-01

**Authors:** Giulia Fiorini, Massimo Rugge, Nimish Vakil, Antonietta D’Errico, Matteo Pavoni, Stefano Guzzinati, Angelo Zullo, Luigi Gatta, Gabriella Massarenti, Beatrice Rosa, Cristina Marchesani, Giulia Collatuzzo, Cinzia Papadia, Giovanni Barbara, Claudio Borghi, Dino Vaira

**Affiliations:** 1 Cardiovascular Medicine Unit, IRCCS Azienda Ospedaliero-Universitaria di Bologna, University of Bologna, Bologna, Italy; 2 Department of Medicine-DIMED, Anatomic Pathology & Cytopathology Unit, University of Padova, 35100 Padova, Italy; 3 Department of Medicine, University of Wisconsin School of Medicine and Public Health, 1685 Highland Ave, Madison, WI 53792 USA; 4 Department of Medical and Surgical Sciences, University of Bologna, Bologna, Italy; 5 Pathology Unit, IRCCS Azienda Ospedaliero-Universitaria di Bologna, Bologna, Italy; 6 Azienda Zero, Veneto Cancer Registry - RTV, Padova, Italy; 7 Gastroenterology Unit, Nuovo Regina Margherita Hospital, Rome, Italy; 8 Dipartimento Staff della Direzione, U.O:C. Governo della Domanda Ambulatoriale e della Diagnostica, Azienda USL Toscana Nord-Ovest , Carrara, Italy; 9 Department of Gastroenterology, Queen Mary University of London, London, UK; 10 Whipps Cross , University Hospital, London, UK; 11 IRCCS Azienda Ospedaliero-Universitaria di Bologna, Bologna, Italy; 12 Department of Biomedical and Clinical Sciences (DIBIC), University of Milan, Milano, Italy

**Keywords:** Gastric cancer, Gastritis, Cancer prevention, *Helicobacter pylori*, BREATH test, OLGA *staging*, EGD

## Abstract

**Background:**

In dyspeptic patients without alarm symptoms, the “Test-and-Treat” (TT) strategy aims to alleviate symptoms and prevent adverse outcomes. An alternative workup expands the TT-strategy by incorporating esophagogastroduodenoscopy (Test-Treat-and-Scope [TT-S]).

**Objective:**

This prospective cross-sectional study provides evidence-based insights into the clinical workup of dyspepsia by applying the TT and TT-S options.

**Design:**

In a consecutive cohort of 2171 dyspeptic patients without alarm symptoms, *H. pylori (Hp)* status was assessed by ^13^C-Urea Breath Test, Rapid urease test, and histology. Histology profiling was based on six biopsies and included atrophy staging (OLGA-system).

**Results:**

Among *Hp*-positive and *Hp*-negative patients, the prevalence of mucosal atrophy was 22.2% and 1.3% (*p* < 0.001), respectively. In the study population, 92.5% did not exhibit atrophic disease (OLGA stage 0). Among *Hp*-positive patients, mucosal atrophy occurred in 142/640 subjects. Nine in 640 (1.4%) *Hp*-positive patients presented advanced atrophy (OLGA-stages III-IV), considered at high-risk for gastric cancer. The overall prevalence of high-risk OLGA stages (III-IV) was below 1.5% (all *Hp*-positive). The mean age of patients with OLGA stages III-IV was 15 years older than that of non-atrophic patients (*p* < 0.0001). In all atrophic OLGA-stages, *Hp-*positive subjects dominated over the *Hp*-negative (test-for-trend; *p* < 0.0001). None of the *Hp*-negative subjects showed extensive atrophy (OLGA-stages III-IV).

**Conclusion:**

In the considered epidemiological context, advanced gastric atrophy (OLGA-stages III-IV), consistently recognized as at risk of cancer development, only occurred in *Hp-*positive patients over 55 years. These results support the priority of the TT-S-strategy in *Hp*-positive patients older than 55, even in the absence of alarm symptoms.

## Introduction

Dyspepsia includes a heterogeneous spectrum of upper abdominal-centered subjective symptoms. Among general and endoscopy populations, the prevalence of dyspepsia varies according to its clinical definitions [1, 2]. Organic dyspepsia coexists with endoscopically/histologically detectable anatomic lesions, including inflammatory diseases, erosions/ulcers, early and advanced pre-malignant, and malignant diseases. Functional dyspepsia refers to a symptomatic condition without identifiable organic disease [3, 4]. The prevalence of functional *versus* organic dyspepsia is biased by the subjective perception of symptoms and the epidemiological prevalence of organic etiologies. The clinical profile of dyspeptic patients with no alarm symptoms (e.g., family history of gastric cancer, unintentional weight loss, hematemesis, progressive dysphagia or odynophagia, recurrent vomiting, and laboratory signs of gastrointestinal bleeding) does not significantly differ in functional and organic cases. However, this distinction has a significant impact on patient management, affecting both therapeutic choices and patient follow-up [5].

*Helicobacter pylori* (*H. pylori*) infection causes chronic, non-self-limiting inflammation and atrophy of the gastric mucosa, impaired stomach function, gastric dysbiosis, and a genotoxic microenvironment. The bacterial infection, especially its more virulent strains, is a known cause of dyspepsia and the leading cause of sporadic gastric cancer (GC) [6–9]. In *H. pylori*-positive patients with dyspepsia, bacterial eradication can successfully treat gastric symptoms and reduce the risk of inflammation-associated GC.

In populations with a high prevalence of *H. pylori* infection, the ‘Test and Treat’ (TT) strategy aims to alleviate symptoms and prevent adverse outcomes associated with the disease. In line with its primary aim, this ‘basic’ workup excludes additional diagnostic procedures, thereby expanding the population that can benefit from immediate bacterial eradication. One disadvantage of the ‘test and treat’ strategy is that it fails to detect gastric precancerous or cancerous lesions in *H. pylori*-infected patients whose symptoms resolve following eradication therapy [10–29].

An alternative clinical workup of *H. pylori*-associated dyspepsia expands the Test-and-Treat strategy by adding esophagogastroduodenoscopy (EGD). This combination of non-invasive and invasive procedures (Test-Treat-and-Scope [TT-S]) enhances diagnostic accuracy, enabling the detection of precancerous lesions that significantly impact patient management [30–32]. Furthermore, this approach allows for the collection of biological samples to test for antibiotic resistance and prevent the use of ineffective medical treatments. Limited access to endoscopy and biopsy procedures, as well as their associated costs, may pose a significant challenge to the routine implementation of the Test-Treat and Scope approach [10–26, 28, 29].

Adjunctive factors supporting the choice of the dyspepsia workup include the epidemiological context (e.g., the country’s demographics, *H. pylori* prevalence and virulence, and the country-specific gastric cancer risk), the healthcare system (e.g., whether it is public or private and the investment in cancer screening programs), the country’s gross domestic product, and patients’ expectancies. All such variables may affect both the Guidelines and the experts’ recommendations [33–37].

In a consecutive cohort of 2171 dyspeptic patients with no alarm symptoms, this study aims to provide evidence-based information on the clinical management of dyspepsia by applying the TT and TT-S options.

## Patients & methods

### Epidemiological setting of the study and criteria for patient enrollment

This prospective cross-sectional cohort study was conducted in an Italian region (Emilia-Romagna), where the prevalence of *H. pylori* infection in the endoscopy population ranges from 25 to 35% in urban and suburban areas. In 2018, there were 795 deaths due to gastric cancer in this region, accounting for 1.6% of all deaths and 5.6% of cancer-related deaths.

Between January 2022 and August 2024, 2213 consecutive patients were referred by their physician to the Digestive Endoscopy Unit (IRCCS Azienda Ospedaliero-Universitaria di Bologna) for dyspepsia with no alarm symptoms.

From the original consecutive cohort of 2213 subjects, the following 42 individuals were excluded based on the following criteria:

- Age younger than 18 or older than 90 years (4 patients);
- Presence of alarm symptoms (family history of GC, unintentional weight loss, hematemesis, progressive dysphagia or odynophagia, recurrent vomiting, and laboratory signs of gastrointestinal bleeding) (2 patients);
- Antisecretory therapies and/or antibiotics within the previous 2 weeks since esophago-gastro-duodenoscopy (EGD) (8 patients);
- Previous *H. pylori* successful eradication (6 patients);
- Previous gastric surgery or endoscopic mucosal resection/dissection (2 patients);
- Biopsy sampling protocol inconsistent with the gastritis staging (two biopsy specimens from the antral mucosa, one from the angularis incisura, and two from oxyntic mucosa: 2 patients);
- Serological testing indicating celiac disease or gastric autoimmune diseases (e.g., positive anti-parietal cell or intrinsic factor antibodies, elevated gastrin, low vitamin B12) (5 patients);
- Declined to participate in the study (13 patients).

### Esophago-gastro-duodenoscopy (EGD)

After initial testing for *H. pylori* infection (13 C-urea breath test; see below), all patients underwent EGD (Olympus→ GIF-100 gastroscope; Japan) regardless of their *H. pylori* status.

An experienced gastroenterologist (DV) performed the endoscopy and biopsy sampling (submitted by the gastric compartment).

The endoscopy features were categorized based on the dominant findings (no focal lesions *versus* erosions or ulcers, either active or healed scars). The location of erosions or ulcers was also recorded, distinguishing between duodenal and distal gastric mucosa down to the *incisura angularis* from proximal lesions. Six biopsy samples were collected from each patient: two from the distal mucous-secreting gastric antrum, two from the antral-oxyntic border at the *incisura angularis*, and two from the gastric oxyntic corpus/ fundus mucosa. The tissue specimens were placed in two vials, distinguishing antral/angular from oxyntic biopsy samples. Two additional biopsy samples were obtained from the gastric antrum for rapid urease test.

### ^13^C-urea breath test (UBT)

^13^C-urea breath test (UBT) used the Expirobacter kit from Sofar Alfasigma S.p.A in Bologna, Italy, according to the manufacturer’s instructions. A test was considered positive if DOB was at least 5‰.

### Rapid urease test (RUT)

Rapid urease test ([RUT], UFT-300 kit Healthcare S.r.l., Milan, Italy). The tissue specimen was immersed in a liquid containing red phenol and urea. The change in the liquid’s color (from yellow to magenta) indicated a positive result.

### Pathology

**Fig. 1:**
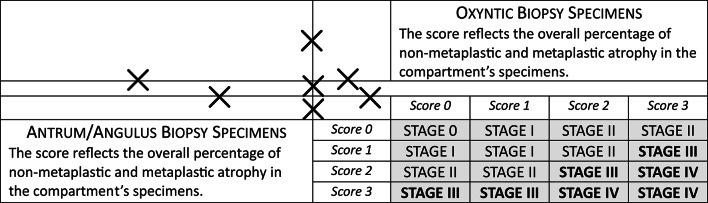
OLGA (Operative Link on Gastritis Assessment) Staging System for gastric mucosa atrophy. The combination of the score values obtained from the two gastric compartments results in the atrophy stage. Score values for atrophy to be applied in mucous-secreting/antral (including *angulus*) and oxyntic/corpus-fundus mucosa: *Score 0* = no atrophy in the specimens obtained from the same compartment; *Score 1*: atrophy (non-metaplastic and metaplastic) involving 1–30% of the specimens obtained from the same compartment; *Score 2*: atrophy (non-metaplastic and metaplastic) involving 30–60% of the specimens obtained from the same compartment; *Score 3*: atrophy (non-metaplastic and metaplastic) involving > 60% of the specimens obtained from the same compartment. The stages considered high-risk for gastric cancer are highlighted in bold

Biopsy specimens were fixed in formalin (5% dilution) and embedded in paraffin. Hematoxylin-eosin (H&E) staining was performed on 4 to 6-micron-thick histological sections. The *H. pylori* status was histologically assessed using a modified Giemsa stain; immunohistochemistry for *H. pylori* was applied when necessary. Two experienced pathologists (AD, LT) jointly evaluated the biopsy sets based on internationally validated criteria. The histological assessment always included the OLGA staging system, which combines the atrophy scores assessed in the antral and oxyntic stomach compartments [38–41]. Figure 1 summarizes the criteria for OLGA staging as proposed in its original publication.

### Assessment of the *H. pylori* status

In all patients, *H. pylori* status was assessed by three methods: (a)^13^C-Urea Breath Test (UBT), (b) Rapid urease test (RUT), (c) histology. *H. pylori*-positive status was based on histology and either a positive UBT or RUT. In discordant results, at least two concordant tests were required to confirm the patient’s infection with *H. pylori*.

### Ethical approval

The study was conducted according to the Declaration of Helsinki and approved by the local Ethics Committee (Protocol No. 47/2012/OSS). Before enrolment, all participants gave written informed consent. Patients or the public were not involved in the design, or conduct, or reporting, or dissemination plans of our research.

### Statistics

The Cochran-Armitage Test was used to identify trends among the proportion of *H. pylori*-positive patients and subjects older than 55 over the different OLGA stages. The Kruskal-Wallis non-parametric test compared the mean age among OLGA stages. To compare the associations of OLGA stage II *versus* stages 0-I with *H. pylori* status, Odds Ratios (OR) were calculated by fitting a multivariable logistic regression model to the data, adjusting for sex, age classes, nationality, and smoking. This calculation excluded seven patients with OLGA stage III and two with OLGA stage IV because they all tested positive for *H. pylori* status, leading to model convergence failure. The SAS EG v.7.15 (SAS Institute Inc., Cary, NC, USA) statistical package was used for all analyses. All statistical tests were two-tailed. A *p*-value < 0.05 was considered statistically significant.

## Results

**Fig. 2:**
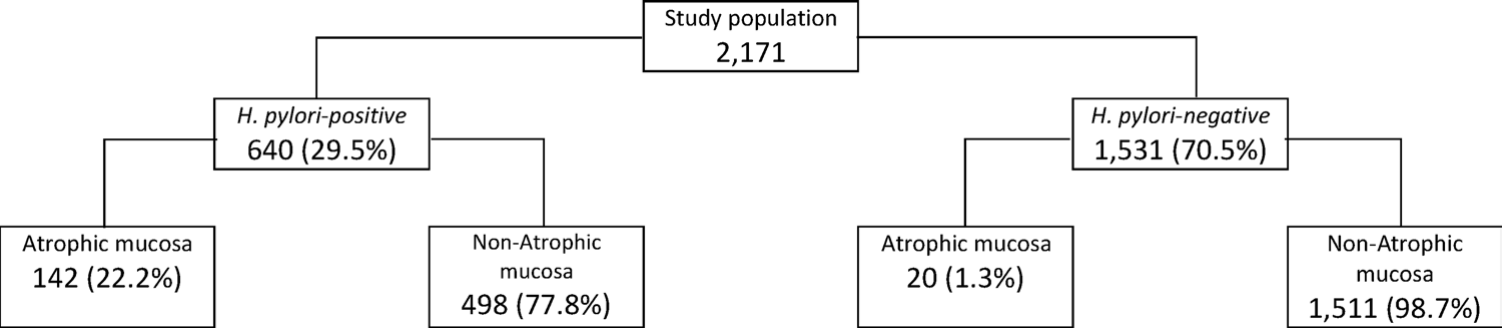
Study population by *H. pylori* status and gastric mucosa histology phenotyping at the enrollment

Figure 2 presents the study population of 2171 individuals, by their *H. pylori* status and histological mucosa phenotyping. The overall prevalence of bacterial infection was 29.5%. Among *H. pylori*-positive and *H. pylori*-negative patients, the prevalence of gastric mucosal atrophy was 22.2% and 1.3% (*p* < 0.001), respectively.

### Study population: demographics, *Helicobacter pylori* status, endoscopy, and histological findings

Table 1 displays demographics, smoking habits, and the endoscopy and histological phenotype of all 2171 individuals by age groups (< 55 *versus* ≥ 55) and *H. pylori* status.

The patient cohort included 577 males and 1394 females (M: F = 0.58), with no sex prevalence by *H. pylori* status (*p* = 0.07). The prevalence of *H. pylori*-negative dyspeptic subjects (70.5%) was higher than that of *H. pylori*-positive patients (25.5%). *H. pylori*-negative subjects significantly prevailed in the Italian native population (*p* = 0.0001). No significant difference emerged between *H. pylori* status and smoking habits (*p* = 0.45).

All erosions/ulcers were found in the *H. pylori*-positive group (overall prevalence 42/640: 10/640 in the pre-pyloric/duodenal areas, and 32/640 in the proximal stomach, including the mucus-secreting oxyntic border along the lesser curvature). Only one *H. pylori*-negative patient had focal lesions (erosion). Among *H. pylori*-positive individuals, erosive/ulcer lesions were significantly linked to age over 55 (*p* < 0.0001).

In the study population, 2009 out of 2171 subjects (92.5%) did not show atrophic disease (OLGA stage 0).

Among *H. pylori*-positive patients, histological phenotyping of the gastric mucosa documented mucosal atrophy in 142 out of 640 subjects. Atrophic lesions only involved the antral mucosa in 106 subjects (74.6%). In 36/142 (25.3%) patients, low-grade (i.e.: G1) atrophic lesions were also documented in oxyntic specimens. The combination of antral and oxyntic atrophy scores resulted in 9/640 (1.4%) *H. pylori*-positive patients harboring high-risk OLGA stages (stage III-IV).

Among *H. pylori*-negative patients, 20/1531 (1.3%) featured mucosal atrophy. Low-grade (i.e.: G1) atrophic lesions always involved the antral compartment; in 4/20 patients, low-grade (i.e.: G1) atrophy also involved oxyntic mucosa.

The overall prevalence of high-risk gastritis stages (OLGA stages III-IV = 9/640) was below 1.5% and all these clustered in the *H. pylori* positive group. The mean age of patients with high-risk gastritis stages (OLGA stages III-IV) was more than 15 years older than that of patients without atrophic lesions (*p* < 0.0001). In all atrophic OLGA stages (I-II-III-IV), *H. pylori-*positive subjects dominated over the *H. pylori*-negative subjects (stage I = 83.9%; stage II = 97.1%; stages III and IV = 100%) (test for trend; *p* < 0.0001).

None of the *H. pylori*-negative subjects showed high-risk OLGA stages (stage III-IV).

**Table 1:** Characteristics of* H. pylori *positive and negative patients. y = years; EGD = esophagogastroduodenoscopy

Considered variables	640 *H. pylori* positive patients		1531 *H. pylori *negative patients	
Male/Female (M: F) *P* value = 0.0701	248/392 (M: F = 1.58)		529/1002 (M: F = 1.89)	
Country of birth: Italy/outside of Italy*P* value = 0.0001	488/152		1383/148	
Smokers/Non-smokers *P* value = 0.4511	131/509		288/1243	
Age	Mean age (63.9)		Mean age (55.8)	
	**Age < 55 y = 290**	**Age ≥ 55 y = 350**	**Age < 55 y = 654**	**Age ≥ 55 y = 877**
EGD				
No focal lesions (erythematous or edematous mucosa, punctate erosions)	**277/290** 95.5% (95% CI 93.1–97.6)	**324/350** 92.6% (95% CI 89.7–95.1)	**654/654** 100% (95% CI 0.0–100)	**876/877** 99.9% (95% CI 99.7–100)
Pre-pyloric/Duodenal (ulcers/erosions)	**3/290** 1.0% (95% CI 0.0–2.4)	**7/350** 2.0% (95% CI 0.6–3.7)	**0/654** 0% (95% CI 0.0-0.6)	**0/877** 0% (95% CI 0.0-0.3)
Angulus/Corpus (ulcers/erosions)	**10/290** 3.4% (95% CI 1.4–5.5)	**19/350** 5.4% (95% CI3.1–8.0)	**0/654** 0% (95% CI 0.0-0.6)	**1/877** 0.1% (95% CI 0.0-0.3)
Histology				
OLGA staging 3–4 (n)	**(0)**	**(9)** Mean age: 68.4	**(0)**	**(0)**

Age is express as mean and categorised in two subgroups (bold) according to the treshold of 55 years. EGD results and OLGA staging are showned according to the aforementioned subgroups (bold)

Years and EGD explanation sholud be moved at the bottom of the table

### Risk factors for high-risk gastritis stages

Using the mean age of the population (55 years), a significant correlation was found between increasing age and increased values of the atrophy stages (OLGA 0 = 25%; OLGA I = 75%; OLGA II = 86%; OLGA III = 100%; OLGA IV = 100%; Cochran-Armitage Trend Test: *p* < 0.0001). Univariate analysis evaluated the risk of having high-stage OLGA (i.e., OLGA stages III-IV, considered high-risk for GC) based on sex, age, country of birth, smoking habits, and *H. pylori* status. A significantly increased risk emerged only for increasing age (as a continuous variable; OR = 1.084; 95% CI 1.024–1.160). There was no significant increased risk associated with sex (OR = 1.438; 95% CI 0.355–5.447) or country of birth (OR = 0.779; 95% CI 0.042–4.267). Based on the evidence that all nine cases of advanced atrophic gastritis (OLGA stage III and IV) occurred in *H. pylori*-positive patients, these cases were excluded from a second analysis focusing on the association between *H. pylori* infection and the risk of developing stage II atrophy, the most advanced stage among the low-risk stages (OLGA 0–II). A multivariable logistic regression model - adjusted for sex, age (as a continuous variable), smoking status, and country of birth - showed a significantly higher risk of OLGA stage II among *H. pylori*-positive patients compared to those with stage 0 or I (OR = 95.2; 95% CI 20.350 to > 999.999) (Table 2). The model also indicated that each additional year of age increased the risk of OLGA progression by approximately 7% (OR = 1.07; 95% CI 1.038–1.107). Given the strong influence of both *H. pylori* positive status and advancing age on progression to stage II, a second logistic model was used to estimate the impact of age as a categorical variable (< 55 *versus* ≥ 55 years). The model predicted a probability of 8.5% for OLGA stage II in patients aged 55 or older, compared with 1.7% in younger patients.

**Table 2:** Multivariable logistic regression model for the low-risk stage II by sex, age, country of birth, smoking habit, and *H. pylori* status

Effect	Odd ratio	95% Confidence intervals
Male versus Female	1.470	0.68–2.83
*H. pylori*-positive versus *H. pylori*-negative	95.192	20.35-999.99
Smokers versus non-smokers	1.397	0.59–3.01
Age (continue)	1.070	1.03–1.10
Born in Italy versus born other countries	1.016	0.32–2.63

## Discussion

In real-world clinical practice, dyspepsia ranks among the most common indications for endoscopy/biopsy procedures [42, 43]. The subjective perception of symptoms and the variable proportion of functional or organic dyspepsia etiology challenge the choice of its clinical work-up [44, 45].

*H. pylori* is a leading cause of dyspepsia [46]. The prevalence of bacterial infection, its virulence, and associated gastric and extra-gastric lesions vary significantly by epidemiological context [47–50]. In countries where *H. pylori*-associated dyspepsia is common and access to endoscopy is limited, the test-and-treat approach aims to identify and eliminate the causative agent, ultimately reducing gastric cancer risk [51, 52]. In regions with low *H. pylori* prevalence, the small number of dyspeptic patients testing positive for *H. pylori* could benefit from an endoscopy approach that involves detecting at-risk lesions. This choice also allows for collecting samples to tailor antibiotic treatment accordingly [53].

These approaches differ significantly in method, cost, clinical yield, and applicability in different epidemiological and healthcare contexts [54]. In Asian countries with a high prevalence of *H. pylori-*related gastric cancer, a more “vigilant” strategy is employed [55]. In a recent meta-analysis including 81 studies from 19 Asian countries, the pooled prevalence of early precancerous gastric lesions was 26.1% (95% CI 22.7–30.0) for chronic atrophic gastritis (CAG) and 22.9% (95%CI 19.7–26.6) for intestinal metaplasia (IM). In the same study, significant odds ratios were found to be associated with *H*. *pylori* infection, with both CAG (2.16; 95% CI, 2.09–2.22) and IM (1.64; 95% CI, 1.57–1.72) being statistically significant. Specifically focusing on a pediatric population, a systematic review and meta-analysis by Abdun and coauthors found that among pediatric *H. pylori*-infected individuals, the overall prevalence of precancerous lesions (i.e., chronic atrophic gastritis with or without IM) was 17.2%. The rates of precancerous lesions in infected individuals were more common in subjects over ten years old and varied significantly across regions, ranging from 33.8% in Africa to 6.3% in Europe. Moreover, in the Chinese pediatric population, more advanced precancerous lesions (i.e., dysplasia) were also identified (1,4%) [56]. This epidemiological evidence strongly supports the rationale for implementing universal endoscopic screening beginning in middle age (TT-S) in populations with a high prevalence of *H. pylori* infection, regardless of patients’ symptoms. In Japan, this strategy is part of the national secondary prevention policy for GC. In a similar epidemiological context, South Korea adopts a proactive approach by implementing culture or molecular testing to guide eradication therapy (Table 3).

**Table 3:** Narrative list of guidelines and expert opinions for the diagnostic/treatment workup of dyspepsia. Treatment refers to eradication in *H.pylori* positive patients

Country/Region	Country specific GC incidence	Clinical presentation	Initial approach	Suggested patients age threshold for TT-S
Thailand (2016) [10]	3.3	No alarm symptoms	Test-and-treat	
		Alarm symptoms	Test-treat and-scope	≥ 45 years
Greece (2020) [11]	6.3	No alarm symptoms	Test-and-treat	
		Alarm symptoms	Test-treat and-scope	≥ 45 years
Japan (2022) [12–14]	27.6	No alarm symptoms	Test-and-treat	
		Alarm symptoms	Test-treat and-scope	≥ 40 years
Australia (2022) [15, 16]	5.3	No alarm symptoms	Test-and-treat	
		Alarm symptoms	Test-treat and-scope	≥ 45 years
UK (2022) [17]	3.6	No alarm symptoms	Test-and-treat	
		Alarm symptoms	Test-treat and-scope	≥ 55 years
Vietnam (2024) [18, 19]	10.3–13.7	No alarm symptoms	Test-and-treat	
		Alarm symptoms	Test-treat and-scope	≥ 40 years (men) ≥35 years (women)
Cile (2024) [20]	14.2	No alarm symptoms	Test-and-treat	
		Alarm symptoms	Test-treat and-scope	≥ 45 years
USA & Canada (2024) [12, 21]	4.3 and 4.7	No alarm symptoms	Test-and-treat	
		Alarm symptoms Therapy failure Symptoms after eradication	Test-treat and-scope	≥ 60 years
Europe (2025) [23–26]	3.3–15.3	No alarm symptoms	Test-and-treat	
		Alarm symptoms	Test-treat and-scope	≥ 50 years
South Korea (2025) [27–29]	27	No alarm symptoms	Test-and-treat	
		Alarm symptoms	Test-treat and-scope	≥ 40 years

GC incidence: **ASR, age standardized ratio ***AGE, recommended starting age for patients undergoing an EDG. Alarm symptoms: unintended weight loss; progressive o new onset of dysphagia; recurrent or persistent vomiting; evidence of GI bleeding; iron-deficiency anemia; family history of gastric cancer; ***Cancer TODAY IARC https://gco.iarc.who.int/today Data version: Globocan 2022 (version 1.1)—08.02.2024. References published in the last decade. The year in the firs column refers to the most recent reference.

The dyspepsia workup differs significantly in Western countries, with *H. pylori* prevalence being considerably lower than in Asian countries. North America (USA and Canada) favors a non-invasive approach (e.g., UBT or stool antigen test) in patients under 60 years without alarm features. Upper endoscopy is reserved for older individuals or those with clinical “red flags”. Meanwhile, based on the British Society of Gastroenterology guidelines, the United Kingdom suggests a test-and-treat strategy in dyspeptic patients under 55 years, with endoscopy indicated for those ≥ 55 years or younger individuals presenting with alarm symptoms [10–29].

This variability in health strategies highlights how risk stratification impacts clinical decision-making in managing dyspepsia, depending on the prevalence of cancer-promoting *H. pylori* infection, resource availability (gross domestic product), public health policies (including population screening programs), and overall population empowerment [51, 57]. Table 3 details the international strategies from the past decade regarding TT *versus* TT-S strategies. When distinguishing between dyspepsia with and without alarm symptoms, the table highlights common recommendations for using the test-and-treat approach for patients who do not show alarm symptoms. The recommended age for the test-and-scope strategy varies significantly, ranging from 35 (Vietnam) to 60 (UK). Except for Thailand, the suggested age reflects each country’s specific incidence rates of gastric cancer (GC). In regions with lower GC incidence, the age cutoff for endoscopy screening is generally older.

The present study, conducted in an epidemiological context with *H. pylori* prevalence below 30%, has associated the infection with a global prevalence of atrophy of less than one-fourth of the considered endoscopy population. Additionally, none of the 1531 dyspeptic patients (of any age) who tested negative for *H. pylori* showed high-risk stages of atrophy. This finding further emphasizes the clinical priority of non-invasive testing for *H. pylori* status in the initial dyspepsia workup.

Univariate and multivariate statistics related to *H. pylori* positive status and increasing age significantly raise the risk of atrophy. In the considered population, the TT-S strategy using a cutoff of 55 years would detect all patients with advanced gastric histology [52]. In *H. pylori-*positive patients, current infection was linked to a stage progression of about 7% per year, providing evidence-based support for timely eradication.

Several factors limit the generalizability of the present results: (a) the country-specific epidemiology of *H. pylori* infection; (b) the healthcare setting of the study; (c) the pragmatic study design, which does not account for dyspeptic patients who were not referred for endoscopy or who declined the procedure; and (d) the single-center project resulting in endoscopic assessments performed by only one endoscopist. Additionally, there is a lack of information regarding the *H. pylori* strains in infected patients, which restricts our understanding of how the bacterium’s virulence influences the progression of atrophy. The strengths of this study include recruiting a large cohort of consecutive outpatients from a real-world gastroenterology practice, utilizing a standardized protocol for gastric biopsy sampling, and internationally consistent histological criteria.

In conclusion, this study highlights the clinical significance of testing *H. pylori* status in the diagnostic approach for patients with dyspepsia. In the considered epidemiological setting, no *H. pylori*-negative dyspeptic patients without alarm symptoms showed at-risk lesions. The potential occurrence of advanced atrophy in *H. pylori*-positive patients over 55 years underscores the importance of test-treat and scope, even in subjects without alarm symptoms.
